# Peptidoglycan hydrolases-potential weapons against *Staphylococcus aureus*

**DOI:** 10.1007/s00253-012-4484-3

**Published:** 2012-10-18

**Authors:** Piotr Szweda, Marta Schielmann, Roman Kotlowski, Grzegorz Gorczyca, Magdalena Zalewska, Slawomir Milewski

**Affiliations:** 1 Department of Pharmaceutical Technology and Biochemistry, Faculty of Chemistry, Gdańsk University of Technology, ul. G. Narutowicza 11/12, 80-233 Gdańsk, Poland; 2 Department of Microbiology, Faculty of Chemistry, Gdańsk University of Technology, ul. G. Narutowicza 11/12, 80-233 Gdańsk, Poland

**Keywords:** *Staphylococcus*, *Staphylococcus aureus*, Antibiotic resistance, Peptidoglycan hydrolases

## Abstract

Bacteria of the genus *Staphylococcus* are common pathogens responsible for a broad spectrum of human and animal infections and belong to the most important etiological factors causing food poisoning. Because of rapid increase in the prevalence of isolation of staphylococci resistant to many antibiotics, there is an urgent need for the development of new alternative chemotherapeutics. A number of studies have recently demonstrated the strong potential of peptidoglycan hydrolases (PHs) to control and treat infections caused by this group of bacteria. PHs cause rapid lysis and death of bacterial cells. The review concentrates on enzymes hydrolyzing peptidoglycan of staphylococci. Usually, they are characterized by high specificity to only *Staphylococcus aureus* cell wall components; however, some of them are also able to lyse cells of other staphylococci, e.g., *Staphylococcus epidermidis*-human pathogen of growing importance and also other groups of bacteria. Some PHs strengthen the bactericidal or bacteriostatic activity of common antibiotics, and as a result, they should be considered as component of combined therapy which could definitely reduced the development of bacterial resistance to both enzymes and antibiotics. The preliminary research revealed that most of these enzymes can be produced using heterologous, especially *Escherichia coli* expression systems; however, still much effort is required to develop more efficient and large-scale production technologies. This review discusses current state on knowledge with emphasis on the possibilities of application of PHs in the context of therapeutics for infections caused by staphylococci.

## Introduction

Bacteria of the *Staphylococcus* genus belong to the most important human and animal pathogens. They are responsible for a broad spectrum of diseases: skin and ocular infections, food poisoning, pneumonia, meningitis, endocarditis, and osteomyelitis. Its high pathogenicity is based on production of a wide array of virulence factors such as protein A, coagulase, collagenase, hyaluronidase, hemolysins, lipases, different toxins, adhesive proteins, and also proteins affecting the biofilm formation. Additionally, *Staphylococcus aureus* has developed several mechanisms that enable the pathogen’s escape from protective immune responses of infected human or animal organisms. Among them, protein A (SpA), staphylokinase, and staphylococcal binder of immunoglobulin are the most important and the best characterized (Kim et al. 2011). The detailed mechanisms of evasion of immune defense by *S*. *aureus* are presented in several excellent review articles (Lambris et al. 2008; Laarman et al. 2010; Langley et al. 2010).

*S*. *aureus* infections are especially difficult to treat because of the evolved resistance to antimicrobial drugs. As rapidly as new antibiotics are introduced, staphylococci have developed efficient mechanisms to neutralize them. In 1928, the Scottish microbiologist Fleming discovered penicillin which was introduced into clinical practice in the early 1940s. However, in 1942, penicillin-resistant staphylococci were recognized, first in hospitals and subsequently in the community, and by the late 1960s, more than 80 % of both community- and hospital-acquired staphylococcal isolates were resistant to penicillin (Lowy 2003; Klein et al. 2007). To overcome infections caused by β-lactamase-producing *S*. *aureus*, a team of scientists from the UK-based pharmaceutical company Beecham produced in 1959 a new, semisynthetic, narrow spectrum, penicillinase-resistant antibiotic called methicillin. However, in 1960, Jevons and coworkers analyzing the group of 5,440 *S*. *aureus* isolates from one hospital-Colindale (London, UK)-found three strains resistant to methicillin (Jevons et al. 1963). Soon after that isolation, *S*. *aureus* strains resistant to this antibiotic have been reported in other countries including Turkey (Cetin and Ang 1962), India (Pal and Ghosh Ray 1964), and Poland (Borowski et al. 1964), where methicillin or other penicillinase-resistant penicillins were not yet available (Ayliffe 1997). Methicillin resistance is associated with the acquisition of a particular resistance island-staphylococcal cassette chromosome *mec* (*SCCmec*). The most important element of *SCCmec* is a gene *mecA* coding for β-lactam-insensitive enzyme of cell wall synthesis called penicillin binding protein 2a or 2′ (PBP 2a or PBP 2′), a 78-kDa protein with low affinity for β-lactam antibiotics. The methicillin-resistant *S*. *aureus* (MRSA) strains are very often classified as multidrug resistant (MDR), which is a consequence of incorporation of many other determinants of resistance into the sequence of *SCCmec*, which acts as a hotspot of integration for genetic mobile elements such as plasmids, transposons, and insertion sequences (Lowy 2003; Stefani and Goglio 2010).

Initially, MRSA strains were encountered only in hospitals and are called *Healthcare Associated* MRSA (HA-MRSA). In the early 1990s, the “new group” of MRSA called community-acquired (CA-MRSA) was identified in indigenous populations in Western Australia. By the PFGE analysis, it was shown that these strains were unrelated to hospital clones of MRSA isolated previously in Australia. In contrast to typical *HA*-*MRSA*, they were susceptible to most antibiotics other than β-lactams but exhibited an unexpected virulence potential (Udo et al. 1993). In the USA, the first well-documented cases of MRSA infection that were truly community-associated occurred in otherwise healthy children in 1997. These children had no risk factors for developing MRSA and all died of severe infection, suggesting that these CA-MRSA strains were especially virulent. Like their Australian counterparts, these CA-MRSA isolates were unrelated to hospital-associated clones and were susceptible to most antibiotics (CDC 1999; Chambers and DeLeo 2009). The staphylococci of both *HA* and *CA*-*MRSA* groups have spread rapidly all over the world and are a serious clinical problem. Recent figures show that infections due to MRSA are responsible for 50 % of hospital-acquired infections with increasing morbidity and mortality, and in some areas, more than 50 % of *S*. *aureus* bacteremias are caused by MRSA (Taiwo 2009). The level of importance of infections caused by staphylococci has been presented by Klein et al. (2007). According to these authors, a number of *S*. *aureus*-related hospitalizations in the USA in the period of time from 1999 to 2005 increased by 62 %, from 294,570 to 477,927, and the estimated number of MRSA-related hospitalizations increased more than two times from 127,036 to 278,203. The authors also noticed that in 2005 there were about 11,400 deaths as a result of *S*. *aureus* infection, of which about 6,640 were MRSA-related (Klein et al. 2007). As it was mentioned above, MRSA strains often represent the MDR phenotype, but the most worrisome problem is the appearance of strains exhibiting resistance or reduced susceptibility to glycopeptides, considered as a gold standard for MRSA therapy. Three classes of vancomycin-resistant *S*. *aureus* that differ in vancomycin susceptibilities have emerged: vancomycin-intermediate *S*. *aureus* (VISA), heterogenous vancomycin-intermediate *S*. *aureus* (hVISA), and high-level vancomycin-resistant *S*. *aureus* (VRSA) (Howden et al. 2010). However, so far, only several strains exhibiting fully vancomycin-resistant strains of *S*. *aureus* (VRSA) due to the acquisition of the *vanA* gene from vancomycin-resistant enterococci have been reported in the USA (Perichon and Courvalin 2009), Iran (Aligholi et al. 2008), and India (Saha et al. 2008). The isolation of VISA and hVISA seems to be a serious emerging problem. In contrast to VRSA, the strains with reduced susceptibility to glycopeptides exhibit high spreading potential. After the first reports of VISA and hVISA from Japan (Hiramatsu et al. 1997), it did not take long for this resistance phenotype to be recognized around the world (Howden et al. 2010).

The problem of staphylococci-related infections is additionally enhanced by the staphylococcal ability of biofilm formation. Biofilm is a structured community of bacterial cells enclosed in a self-produced polymeric matrix and adherent to an inert or living surface (Costerton et al. 1999). Microorganisms in biofilms are evidently less susceptible to antibiotics, importantly due to altered growth rate and delayed penetration of antimicrobial agents within the biofilm structure (Melchior et al. 2006, 2007). Some of the previous studies proved that the biofilm-forming bacteria may be in fact 10–1,000 times more resistant to antimicrobial agents than the same cells growing in a planktonic manner as it takes place during the in vitro assays of antibiotic activity (Amorena et al. 1999; Melchior et al. 2006, 2007). Antibiotic resistance of biofilms often leads to the failure of conventional antibiotic therapy and recurrence of infections.

*S*. *aureus* and other bacteria of this genus, including MRSA, are also important animal pathogens. The number of publications presenting isolation of strains resistant to methicillin from animal sources has increased evidentially in the last few years (Weese and van Duijkeren 2010; Loeffler and Lloyd 2010). Staphylococci are also an important problem for food technologists especially in the meat, dairy, and fish industries (Vanderhaeghen et al. 2010).

The evident increase in the antibiotic resistance of staphylococci, one of the most common and dangerous bacterial pathogens, observed worldwide, stimulates the development of new alternative, effective, and inexpensive agents for the treatment of this group of microorganism. Some of the alternative therapies in *S*. *aureus* diseases, such as bacteriophages, antimicrobial peptides, plant- (e.g., stilbenoids and flavonoids) and animal-derived (e.g., chitosan and propolis) compounds, vaccines, and photodynamic therapy have been recently discussed by Kurlenda and Grinholc (2012). The present paper is devoted to discuss the possibilities of the prevention and treatment of staphylococcal infections by using peptidoglycan hydrolases (PHs), enzymes exhibiting the ability to hydrolyze bacterial cell wall components. In our opinion, these enzymes constitute one of the most promising but still underestimated groups of potential antistaphylococcal chemotherapeutics.

## Cell wall structure of *S*. *aureus*

**Fig. 1:**
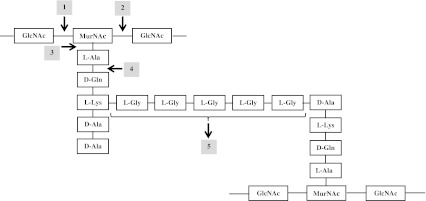
The structure of *S*. *aureus* peptidoglycan (according to Fenton et al. 2010a, with some modifications), the *arrows* indicate the cleavage sites: *1 N*-acetyl muramidase, *2 N*-acetyl-β-d-glucosaminidase, *3 N*-acetylmuramoyl-l-alanine amidase, *4* l-alanoyl-d-glutamate endopeptidases, and *5* interpeptide bridge endopeptidases (e.g., lysostaphin). Abbreviations: *GlcNAc N*-acetylglucosamine, *MurNAc N*-acetylmuramic acid

*S*. *aureus* is a Gram-positive spherical bacterium approximately 1 μm in diameter. Its cells are surrounded by a murein sacculus. As in the case of other Gram-positive bacteria, the cell wall of staphylococci is composed of teichoic acids, surface proteins, and first of all, the macromolecular net of peptidoglycan. The peptidoglycan is composed of glycan chains made up of the alternating amino sugars *N*-acetylglucosamine and *N*-acetylmuramic acid. Stem pentapeptides (l-Ala-d-iso-Gln-l-Lys-d-Ala-d-Ala) are attached to the carboxyl group of each *N*-acetylmuramic acid, and interpeptide bridges (pentaglycines, made up of glycine residues) connect the lysine component of one stem peptide to the penultimate d-alanine of a neighboring stem peptide in which glycan strands are cross-linked with short peptide motives (Tomasz 2006) (Fig. 1). A major function of the cell wall is to act as a pressure vessel, preventing overexpansion when water enters the cell. The inhibition of activity of enzymes, which participate in the synthesis of peptidoglycan, is the basis of activity of many antibacterial antibiotics such as β-lactams (e.g., penicillins and cephalosporins), glycopeptides (e.g., vancomycin and teicoplanin), fosfomycin, daptomycin, nisin, bacitracin, and many others. The cells of bacteria, especially the Gram-positive bacteria, which are not protected with a properly synthesized cell wall, are not able to survive. The structure of peptidoglycan consists of at least five types of bonds, which are molecular targets for existing in nature bacterial cell wall hydrolyzing enzymes, PHs. The localization of these bonds and their corresponding enzymes is presented in Fig. 1. The targets marked with numbers 1–4 are present in the peptidoglycan of most genus of bacteria, while the pentaglycine interpeptide cross-bridge is a unique component of *S*. *aureus* cell wall and is a specific substrate for lysostaphin, the best known staphylococcal peptidoglycan hydrolase, but also for many other, less known enzymes, which are characterized in this review. The hydrolysis of peptidoglycan by PHs results in a strong bactericidal effect which makes this group of enzymes an alternative antibacterial weapon, which currently is not widely used not only in clinical practice, but also in veterinary and food technology.

In order to protect against activity of cell wall hydrolases, bacteria have specific modifications of the backbone peptidoglycan. Specific modifications like N-deacetylation, N-glycolylation, O-acetylation, or modifications of amino acid sequence of peptide bridges render some bacteria resistant to PHs. This phenomenon should be taken into account in the development of clinical, veterinary, or food application of this group of enzymes (Callewaert et al. 2011).

## Peptidoglycan hydrolases with antistaphylococcal activity

**Fig. 2:**
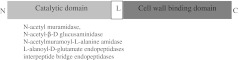
Basic structure of peptidoglycan hydrolases according to Fischetti (2008). N-terminal catalytic domain retains the activity to cleave one of the bonds in the staphylococcal peptidoglycan, some of the enzymes contain two catalytic domains (e.g., AtlE, LysK). Sequence comparison of enzymes in the same enzyme class indicates that the catalytic region is highly conserved, while C-terminal binding domain is highly conserved among most of *S*. *aureus* bactericidal peptidoglycan hydrolases also belonging to different enzyme classes and produced in different hosts (staphylococci, other bacteria, and bacteriophages). *L*—short linker between functional domains

Enzymes of peptidoglycan hydrolase activity are produced by all groups of living organisms and also viruses. In the structure of all of them, two domains can be distinguished: catalytic N-terminal domain and C-terminal cell wall binding domain (Fig. 2). In the case of human, animal, and plant organisms, these enzymes play an important role in the suppression of bacterial infections as a component of innate immune system. The bacteriophage PHs are called lysins or endolysins, and they perform crucial roles in the viral infection cycle of the bacterial host. Interestingly, the enzymes which are capable of hydrolyzing the peptidoglycan of the bacterial cell envelope are ubiquitous also among bacteria and are called autolysins. The bacterial cell wall is a dynamic structure, which is modified strictly according to the “cell cycle.” The autolysins play an important role in cell wall growth, cell separation, cell wall turnover, competence for genetic transformation, flagellum formation, sporulation, and in the lysis of bacteria induced by the β-lactam antibiotics (Shockman and Holtje 1994; Antignac et al. 2007). Below, we have presented the most promising PHs of different origin, which potentially could be used as antistaphylococcal agents.

### Lysozymes

In this paper, the presence of lysozymes can be surprising. In contrast to most Gram-positive bacteria, the pathogenic staphylococci exhibit extremely high resistance to lysozymes of different origin, including the most popular hen egg white lysozyme. It is crucial for their interactions with the host organisms that produce lysozyme as an initial defense against bacterial infection. The lack of susceptibility of the staphylococcal cell wall to lysozyme has a complex background and is still not well understood. Several studies have suggested that it is primarily caused by O-acetylation at position *C*-6 of the *N*-acetylmuramic acid (NAM) residue (Clarke and Dupont 1991; Bera et al. 2005). The studies of Bera et al. (2006) revealed that the presence of the enzyme peptidoglycan *O*-acetyltransferase (OatA) in various staphylococcal species correlates with lysozyme resistance and pathogenicity. The muramic acid was O-acetylated only in pathogenic, lysozyme-resistant staphylococci (e.g., *S*. *aureus*, *S*. *epidermidis*, *Staphylococcus lugdunensis*, and others). All nonpathogenic species were lysozyme sensitive (e.g., *Staphylococcus carnosus*, *Staphylococcus gallinarum*, and *Staphylococcus xylosus*) or hypersensitive (e.g., *Staphylococcus equorum*, *Staphylococcus lentus*, and *Staphylococcus arlettae*) (Bera et al. 2006). It has been also documented that the resistance of staphylococci to lysozymes occurs due to two other important mechanisms. First, it is caused by binding of wall teichoic acids to the C-6 position of NAM (Bera et al. 2007). Second, it is based on extensive cross-linking of their peptidoglycan, which results from the regulated action of penicillin binding protein 4 (Atilano et al. 2010).

Lysozymes have been included in this review because until now the hen egg white lysozyme is one of the few natural antimicrobials at all and the only PH that is approved as a food preservative in EU and several other countries. It has been also successfully applied as a component of “active” food packing materials (Callewaert et al. 2011). However, it does not exhibit activity against staphylococci. Lysozyme is incontestably the most extensively studied PH and can be a good model for future studies of medical and food application of enzymes hydrolyzing staphylococcal cell wall.

### Lysostaphin

Lysostaphin is a zinc metalloproteinase isolated from *Staphylococcus simulans* biovar *staphylolyticus* (Schindler and Schuhardt 1964, 1965; Sloan et al. 1982). It degrades the cell wall of almost all known staphylococcal species. The target of the lysostaphin activity is the pentaglycine interpeptide bridges of the unique staphylococcal peptidoglycan (Schleifer and Kandler 1972; Iversen and Grov 1973). Other Gram-positive and Gram-negative bacteria are not susceptible to this enzyme (Schindler and Schuhardt 1964). The native lysostaphin is organized as a preproprotein containing a signal peptide of 33 amino acid residues, a propeptide of 211 amino acid residues (from which 195 are organized in 15 tandem repeats of 13 amino acids in length), and a mature protein of 246 amino acid residues (Heinrich et al. 1987; Thumm and Gotz 1997). The N-terminal prepeptide (signal peptide) cleaved intracellularly and the propeptide removed extracellularly by a cysteine protease do not participate in the degradation of staphylococcal cells (Zhou et al. 1989). The catalytic activity for the mature 27-kDa lysostaphin showed approximately 4.5-fold higher activity than the precursor protein, and in fact, only this fragment of the enzyme is considered for future applications (Thumm and Gotz 1997). The native producer of the enzyme, *S*. *simulans*, is not susceptible to autolysis because of the expression of the Lif protein (lysostaphin immunity factor), which incorporates the serine residues into the interpeptide bridges at positions 3 and 5 (DeHart et al. 1995; Thumm and Gotz 1997; Ehlert et al. 2000). Incorporation of amino acids other than glycine (predominantly serine and alanine) accounts for the decreased susceptibility of many coagulase-negative staphylococci to lysostaphin, which is unable to hydrolyse Gly-Ser, Ser-Gly, Ala-Gly, and Gly-Ala peptide bonds (Robinson et al. 1979; Kiri et al. 2002). The enzyme has maximum activity at pH 7.5 and about 40 °C (Schindler and Schuhardt 1965) and is inhibited by exogenously added zinc and agents that would chelate endogenous zinc atoms such as *o*-phenanthroline or EDTA (Zygmunt and Tawormina 1972; Park et al. 1995). The unique biological activity of lysostaphin presents numerous possibilities for applications of this enzyme as an antistaphylococcal agent in medicine, veterinary, food industry, and also in research fields.

The presence of pentaglycine interpeptide cross-bridge and its susceptibility to lysostaphin is a characteristic feature of *S*. *aureus*, which is used for specific identification of this genus of bacteria and its differentiation from the closely related *Micrococci*. Because of the previously mentioned unique organization of peptidoglycan, staphylococci are resistant to most conventional lysis methods that use lysozyme, detergents, and ultrasound. Lysostaphin is frequently used as a staphylolytic agent for the liberation of intracellular enzymes, nucleic acids, and cell membrane and surface components.

The enzyme was first described in 1964 (Schindler and Schuhardt 1964), and since its discovery, it was intensively tested as a potential chemotherapeutic agent against staphylococcal infections. In the early 1970s, lysostaphin was first successfully used for the treatment of a staphylococcal abscess in a human neutropenic patient (Stark et al. 1974). The application of the enzyme was also shown to be effective in reducing the nasal carriage of *S*. *aureus* in humans (Harris et al. 1967; Quickel et al. 1971). Because of wide availability of different groups of antibiotics, which were cheaper and easier in production, the trials of application of lysostaphin were stopped for over two decades until the late 1990s. During the last 15 years, there have been numerous reports which definitely confirm the therapeutic potential of lysostaphin in the treatment of infections caused by staphylococci including MRSA and VISA.

Climo et al. (1998) compared the effectiveness of various regimens of intravenous dosing of lysostaphin to that of vancomycin in the rabbit model of aortic valve endocarditis caused by a clinical methicillin-resistant *S*. *aureus* isolate. The reduced susceptibility to vancomycin of the strain was generated through the stepwise passage in the presence of increasing concentrations of glycopeptides. The most active regimen, lysostaphin given three times daily, produced sterile vegetations in 10 of 11 treated rabbits, while the number of bacteria in the untreated controls increased to the level of 8.5 × 10^3^ CFU/g. In contrast, vancomycin given twice daily sterilized no vegetations and reduced vegetation bacterial counts by only 4.8 × 10^3^ CFU/g compared to the counts in the untreated controls. The authors also revealed that the enzyme was well tolerated by the rabbits, and no evidence of immunological reactions following up to 9 weeks of intravenous administration of the lysostaphin was observed. The same researchers confirmed excellent bactericidal activity of lysostaphin for the treatment of experimental aortic valve endocarditis caused by a clinical isolate of VISA (Patron et al. 1999), while in the case of experimental aortic valve endocarditis caused by oxacillin-resistant *S*. *epidermidis*, it was necessary to use a combination of lysostaphin and nafcillin. The combination of these two agents was as effective as vancomycin alone and significantly better than lysostaphin or nafcillin alone (Kiri et al. 2002).

The group of O’Callaghan at LSU Medical Center in New Orleans revealed that lysostaphin is effective in treating keratitis caused by methicillin-sensitive or methicillin-resistant *S*. *aureus* and endophthalmitis mediated by MRSA. Methicillin-resistant *S*. *aureus* strain 301 (MRSA 301) or a methicillin-sensitive strain of low virulence, ISP546, was intrastromally injected into rabbit corneas. Rabbit eyes were treated topically every 30 min from 4 to 9 or 10 to 15 h postinfection with 0.28 % lysostaphin or 5 % vancomycin. With early therapy (4–9 h postinfection), lysostaphin sterilized all MRSA 301-infected corneas, whereas untreated corneas contained 6.52 × 10^3^ CFU/cornea. Corneas infected with MRSA 301 and treated similarly with vancomycin retained 2.3 ± 0.85 × 10^3^ CFU/cornea, and none were sterile. When therapy was begun later (10–15 h postinfection), the residual bacteria in lysostaphin-treated eyes were significantly less numerous than in vancomycin-treated eyes (0.58 ± 0.34 vs. 5.83 ± 0.16 × 10^3^ CFU/cornea), respectively (Dajcs et al. 2000). O’Callaghan and coworkers also tested the possibilities of application of lysostaphin in the treatment of experimental endophthalmitis mediated by coagulase-negative *Staphylococcus* species, which in fact is the leading cause of postsurgical endophthalmitis. As a result of some modifications of the amino acid composition of interpeptide bridge in the peptidoglycan, the coagulase-negative staphylococci are generally more resistant to lysostaphin. Interestingly, the results of the investigation were successful. Treatment with lysostaphin significantly reduced the bacterial count as compared with untreated eyes for 13 strains tested in vivo, and only in the case of two *S*. *haemolyticus* strains, two methicillin-resistant *S*. *epidermidis* (MRSE) strains, and one *Staphylococcus cohnii* strain, the results of treatment were not satisfactory. Treatment with lysostaphin reduced the colony forming units per milliliter of the number of methicillin-sensitive *S*. *epidermidis* strains by 6-logs; for *Staphylococcus warneri*, there was a 2-log reduction; and for the other species, a 4-log reduction in colony forming units per milliliter relative to untreated eyes (McCormick et al. 2006).

An important problem in the antibiotic therapy of staphylococcal infections is the ability of this group of microorganisms to form a biofilm. Some reports have shown that lysostaphin is a promising agent also in the eradication of biofilm of staphylococci from biotic and abiotic surfaces. The investigation carried out by Wu et al. (2003) revealed that lysostaphin not only killed *S*. *aureus* growing in biofilms, but also disrupted the extracellular matrix of *S*. *aureus* biofilms in vitro on plastic and glass surfaces at concentrations as low as 1 μg/mL. In contrast, even high concentrations of oxacillin (400 μg/mL), vancomycin (800 μg/mL), and clindamycin (800 μg/mL) had no effect on the established *S*. *aureus* biofilms in this system, even after 24 h. Higher concentrations of lysostaphin also disrupted *S*. *epidermidis* biofilms. The activity of lysostaphin towards biofilms built by clinical and reference *S*. *aureus* and *S*. *epidermidis* was also confirmed by Walencka et al. (2005). The lysostaphin susceptibility of *Staphylococcus* tested strains of planktonic and biofilm cultures varied and was strain dependent. The authors noticed a synergistic effect of the enzyme and some antibiotics: oxacillin, vancomycin, and linezolid in the eradication of staphylococcal biofilm. The combination of lysostaphin and linezolid was effective in disruption of biofilm formed by MSSA, MRSA, and even hVISA strains. The biofilm of the hVISA strain was resistant to the combination of the enzyme with oxacillin and vancomycin. The resistance towards the combination of lysostaphin and oxacillin was also noticed in the case of biofilm of 6756/99 MRSE strain (Walencka et al. 2005, 2006). Recently, Aguinaga et al. (2011) studied the synergistic biofilm eradication effect of the combination of lysostaphin and a broader spectrum of antibiotics. Lysostaphin combined with cefazolin, clarithromycin, doxycycline, levofloxacin, linezolid, and quinupristin/dalfopristin exhibited a synergistic effect against the MSSA strain in terms of its minimal biofilm eradication concentration (MBEC), with reductions of 2–11-fold dilutions. The synergistic effect of lysostaphin in combination with clarithromycin, doxycycline, levofloxacin, rifampicin, and teicoplanin against the MRSA strain, with 2–14-fold reduction of their MBEC, was also confirmed (Aguinaga et al. 2011). The studies carried out by Biosynexus Incorporated (Gaithersburg) revealed that lysostaphin could also eradicate in vivo established *S*. *aureus* biofilms on implanted jugular vein catheters in mice. The enzyme administered at 15 mg/kg in combination with 50 mg/kg nafcillin three times daily for 4 days eradicated established *S*. *aureus*, including MRSA, biofilms from implanted catheters, and sterilized heart and liver infections of *S*. *aureus*-infected mice. Furthermore, a single pre-instillation of 10 mg/kg lysostaphin in catheters completely protected catheterized mice from a subsequent biofilm infection (Kokai-Kun et al. 2009). In 2003, the Biosynexus Company presented results of another interesting investigation. That study demonstrated that a single application of 0.5 % lysostaphin (actual dose, approximately 150 μg of lysostaphin), formulated in a petrolatum-based cream, dramatically reduces *S*. *aureus* nasal colonization in 100 % of animals tested and eradicates *S*. *aureus* nasal colonization in 93 % of animals in a cotton rat model. A single dose of lysostaphin cream was more effective than a single dose of mupirocin ointment in eradicating *S*. *aureus* nasal colonization in this animal model (Kokai-Kun et al. 2003). Similar results were obtained by Cui et al. (2010), who used chitosan-o/w cream incorporated with lysostaphin. The anterior nares are considered as a primary ecological niche for *S*. *aureus*, and the nasal colonization by this opportunistic pathogen increases the risk of infection development. Clearance of *S*. *aureus* nasal colonization could greatly reduce this risk.

During the last 2 years, a completely new approach of application of lysostaphin in the clinical area has been developed. The enzyme is immobilized or simply adhered on the surface of medically important materials such as meshes which are used in herniorrhaphy or wound dressing. The results of preliminary research are very promising. Miao et al. (2011) immobilized lysostaphin on the surface of cellulose fibers, which were processed to obtain bandage. The resulting material showed high activity against *S*. *aureus* in in vitro skin model with low toxicity toward keratinocytes. The aim of the study carried out by Belyansky et al. (2011) was to determine whether lysostaphin adhered to porcine mesh can impact host survival in the animal model. Animals implanted with lysostaphin containing mesh had a dramatically improved rate of survival. Only 1 out of 30 rats with bacterial inoculum not treated with lysostaphin survived. By comparison, all of the 25 lysostaphin-treated animals were completely cleared of *S*. *aureus* when challenged with bacterial concentrations of 1 × 10^6^ and 1 × 10^8^ with maintenance of mesh integrity for 60 days.

The next interesting proposal of the application of lysostaphin was that of Pangule et al. (2010). The researchers designated and obtained carbon nanotube–lysostaphin conjugates. These enzyme-based composites were highly efficient in killing MRSA (>99 % within 2 h) without release of the enzyme into the solution. Additionally, these films were reusable and stable under dry storage conditions for a month. The authors suggest that the enzyme-based film formulations may be used to prevent growth of staphylococci on various common surfaces in hospital settings.

Staphylococci are also one of the most important pathogens of wild and domestic animals. In recent years, an evident increase in the number of reports in the isolation of MRSA from pigs and calf has been observed. To address this issue, some interesting propositions of the application of lysostaphin in veterinary, especially for the treatment of bovine mastitis, have been presented. Oldham and Daley infected the mammary glands of 30 Holstein-Freisian dairy cattle with *S*. *aureus* in all quarters. Next, the infected animals were injected through the teat canal with a single dose of recombinant lysostaphin (dose response 1 to 500 mg) or after three successive p.m. milkings with 100 mg of recombinant lysostaphin in 60 mL of sterile phosphate-buffered saline. Animals were considered cured if the milk remained free of *S*. *aureus* for a total of 28 milkings after the last treatment. The cure rate of quarters receiving recombinant lysostaphin (100 mg in sterile phosphate-buffered saline, administered over three consecutive p.m. milkings) was 20 % compared with 29 % for sodium cephapirin in saline and 57 % for a commercial antibiotic formulation, respectively (Oldham and Daley 1991). These observations were lately confirmed by the group of Jakubczak et al. (2010). High in vitro bactericidal activity of lysostaphin against *S*. *aureus* isolated from mastitis was recently confirmed by Zhang et al. (2012). However, the most advanced proposition of the application of lysostaphin for the protection of cattle against staphylococcal mastitis was proposed by Wall and coworkers. In 2001, the researchers revealed possibilities of production of biologically active lysostaphin in the mammary glands of mice. The obtained lines of transgenic mice, in which the 5′-flanking region of the ovine β-lactoglobulin gene directed the secretion of lysostaphin into milk, exhibit substantial resistance to an intramammary challenge of 10^4^ CFU of *S*. *aureus*, with the highest expressing line being completely resistant (Kerr et al. 2001). Finally, in 2005, Wall’s group produced transgenic cows secreting lysostaphin at concentrations ranging from 0.9 to 14 μg/mL of their milk. Protection against *S*. *aureus* mastitis appears to be achievable with as little as 3 μg/mL of lysostaphin in milk. The milk’s ability to kill *S*. *aureus* was confirmed by in vitro assays (Wall et al. 2005).

The foodborne illnesses caused by *S*. *aureus* and other *Staphylococcus* species are usually associated with products that cannot be preserved by thermal methods, such as meat, dairy, poultry, egg, and cream-filled bakery products. In this case, the favorable way of pathogen inactivation is application of bacteriocins. Because of the biological activity of lysostaphin, this protein seems to be a very good candidate for a natural food preservative. Unfortunately, very few studies have addressed the preservation of food products against *Staphylococcus* growth by this enzyme application. The gene encoding lysostaphin has been cloned and expressed into *Lactobacillus casei*, *Lactobacillus curvatus*, and *Lactobacillus sake* (Gaier et al. 1992; Cavadini et al. 1996, 1998). The transgenic *L*. *curvatus* producing biologically active enzyme was successfully used for preservation of fermented sausages. In this case, a 90 % reduction of activity of the enzyme was observed, but the residual activity was sufficiently high to reduce *Staphylococcus* counts from 10^4^ to 10^5^ CFU/g within 2 to 3 days to below the level of detection (Cavadini et al. 1998). Also, purified recombinant lysostaphin expressed in *E. coli* cells has been developed for food protection against *S*. *aureus* by Szweda et al. (2007). The population of *S*. *aureus* in milk preserved at 4 °C by lysostaphin added up to concentrations of 1.5 or 3.0 μg/mL was reduced by 0.73 and 0.92 log(CFU/mL) in comparison to control samples without enzyme addition. The protective influence of lysostaphin was diminished in case of milk storage at 20 °C prolonged up to 24 h. Furthermore, a final reduction level by 0.92 log(CFU/mL) was achieved after 24 h of pork storage. The smaller and more dependent on enzyme concentration inactivation of *S*. *aureus* was observed in the case of mayonnaise salads. The presented results cannot be classified as really satisfactory; however, in our opinion, the enzyme has a great potential as a food preservative.

Recently, Desbois and Coote (2011) revealed a strong bactericidal synergy of lysostaphin in combination with some other antimicrobial peptides. It is also highly possible that the bactericidal effect of the enzyme can be enhanced by some food preservatives or natural substances (so far not tested). Some of the combinations of lysostaphin and other agents can be more effective in the eradication of staphylococci from food products. Additionally, resistance of other bacteria against lysostaphin activity makes this enzyme useful especially for the preservation of food containing probiotic microflora. The crucial issue for large-scale application of the enzyme is its availability and low costs of its production. In fact, recently, several efficient heterologous systems of expression of lysostaphin have been developed (Mierau et al. 2005a, b; Szweda et al. 2005; Sharma et al. 2006). However, in our opinion, it is still necessary to make an effort to do scaling up and improve performance of proposed technologies.

### LasA-staphylolysin

In fact, the only bacterial PH that has been tested as a potential staphylolytic agent except lysostaphin is the enzyme LasA also called staphylolysin produced by *Pseudomonas aeruginosa* (Kessler et al. 1993; Kessler and Ohman 2004). LasA is synthesized as an inactive precursor that is composed of a signal peptide (3 kDa), a propeptide (22 kDa), and a mature catalytic domain (20 kDa) (Gustin et al. 1996; Schad and Iglewski 1988). The signal peptide is cleaved as the protein crosses the inner membrane, and the propeptide remains covalently attached until the protein is secreted from the cell, then it is hydrolyzed by other extracellular proteases produced by *P*. *aeruginosa* (Kessler et al. 1998). LasA is implicated in a range of processes related to *Pseudomonas* virulence, including stimulation of ectodomain shedding of the cell surface heparan sulfate proteoglycan syndecan-1 and elastin degradation in connective tissue (Park et al. 2001; Vessillier et al. 2001). Similar to lysostaphin, the enzyme cleaves the pentaglycine bridges of the cell wall of peptidoglycan of *S*. *aureus*. Moreover, LasA is “tolerant” for some modifications of amino acid composition within pentaglycine interpeptide bridge, and in consequence, the enzyme is able to lyse also other types of staphylococci including *Staphylococcus saprophyticus*, *S*. *epidermidis*, and *S*. *warneri* (Brito et al. 1989; Vessillier et al. 2001; Kessler et al. 2004). The detailed catalytic mechanism of this protein including recognition a wider array of substrates has been solved by Spencer et al. (2010) who determined the three-dimensional structure of LasA.

In terms of the application of staphylolysin as a bactericidal agent against staphylococci in animal models, several interesting reports have been presented by the group of Barequet from Tel Aviv University (Israel). The authors revealed that staphylolysin is effective in the treatment of both MSSA and MRSA experimental keratitis. The enzyme completely sterilized the infected rat corneas when treatment was initiated 4 h after infection and reduced the number of viable bacteria (colony forming units) in both MSSA and MRSA-infected corneas by 3–4 orders of magnitude as compared to controls when treatment was initiated 10 h postinfection. Furthermore, staphylolysin was found to be even more effective than vancomycin in eradicating MRSA cells (Barequet et al. 2004). Satisfactory activity of LasA was confirmed in the investigation carried out with rat corneas infected with clinical isolates of MRSA strains. The staphylolysin-treated eyes infected with the MRSA strains were either completely sterilized or showed 3–4 orders of magnitude decrease in the number of CFU/cornea in comparison to control. The histopathological analysis of the methicillin-sensitive (MSSA) strain-infected eyes at 84 h after completion of treatment showed moderate inflammation in the staphylolysin-treated eyes as compared with extensive abscess formation in the control group (Barequet et al. 2012). The same group demonstrated that staphylolysin is effective in the treatment of experimental endophthalmitis in rats caused by MRSA strain. In eyes treated by the single injection regimen, staphylolysin reduced the mean CFU value per vitreous 3-fold as compared to control (2,055 ± 3,144 and 6,432 ± 6,389 CFU/vitreous, respectively; *P* = 0.02). The repeated injection protocol was more effective, reducing the mean CFU value per vitreous by 2 orders of magnitude as compared to control (1,148 ± 3,096 and 143,519 ± 151,358 CFU/vitreous, respectively; *P* = 0.0005). Histopathological analysis showed no structural damage in the eyes injected intravitreally with staphylolysin (Barequet et al. 2009).

Though the results presented above are promising, surprisingly enough, there are no more information about investigations concerning the application of staphylolysin for the treatment of infections caused by *S*. *aureus* and other staphylococci. In comparison to lysostaphin, the LasA represents a broader spectrum of activity and could be tested for example for the treatment of infections caused by *S*. *epidermidis*, an opportunistic pathogen of growing importance. It is also interesting that there is no information about the production of the enzyme using heterologous expression system. In all presented reports, the protein was isolated from *P*. *aeruginosa* culture supernatants. In our opinion, new methods of production and application of LasA are an interesting field for research in the nearest future. It can be assumed that the results of these investigations would lead to many applications of this enzyme.

### Staphylococcal autolysins

As it was mentioned above, PHs are essential for many important processes of modification of bacterial cell wall envelope. In the living cells, they are expressed in a precisely defined amount and at an accurate time. This timing protects bacteria against possible autolysis. On the other hand, several reports revealed that the bacterial cells treated produced by themselves PHs, but taken from the exogenous sources, are killed as a result of hydrolysis of peptidoglycan constituent of their cell wall.

Since the first report on *S*. *aureus* autolysins (Welsch and Salmon 1950), a variety of intracellular and extracellular enzymes hydrolyzing peptidoglycan have been reported and characterized in this bacterium (Sugai 1997). In our opinion, some of them deserve a special attention from the point of view of their application as bactericidal enzymes against staphylococci. According to our best knowledge, so far, studies on this subject have not been performed. This is surprising, especially taking into account the number of investigations of potential applications of lysostaphin or even LasA.

One of the most interesting staphylococcal autolysins is the ALE-1 enzyme produced by *Staphylococcus capitis* EPK1. Its primary structure is very similar to lysostaphin, and the identity of the putative functional domains of the enzymes is 83 % at the amino acid level. Both are glycylglycine endopeptidases and hydrolyse the pentaglycine interpeptide bridges of *S*. *aureus* peptidoglycan. They consist of a multiple repeat N-terminal domain fused to an active site containing a C-terminal substrate (peptidoglycan)-specific domain. The ALE-1 enzyme has been cloned and expressed in *E*. *coli* cells, and its staphylolytic activity was confirmed with heat-killed and viable *S*. *aureus* FDA209P (Sugai et al. 1997). Another interesting glycylglycine endopeptidase autolysin produced by *S*. *aureus* is the enzyme called LytM. Taking into account the putative functional domains, it shows 51 and 50 % identity with lysostaphin and ALE-1, respectively. All these enzymes are members of the Zn^2+^ protease family and contain a homologous 38-amino-acid-long motif, Tyr-X-His-X_11_-Val-X_12/20_-Gly-X_5-6_-His. However, in contrary to the two proteins described earlier, the gene encoding LytM is located on the chromosome, not on the plasmid. It may indicate that LytM plays an important role in actively growing and dividing cells. The enzyme was first described in 1997 (Ramadurai and Jayaswal 1997), and in 1999, it was overexpressed in *E*. *coli* as a fusion protein with a six-histidine residue tag at its N-terminus (Ramadurai et al. 1999). The recombinant protein effectively hydrolyzed peptidoglycan of heat-killed and autoclaved *S*. *aureus* N450 and was completely inactive towards the cell wall of *Micrococcus luteus*, which does not contain a pentaglycine motif in its cell wall. The protein has been crystallized and its active site domains have been mapped (Firczuk et al. 2005; Odintsov et al. 2004). Despite the evident similarity of amino acid sequence, the enzymes are differently affected by various physical and chemical factors. For example, lysostaphin and LytM are strongly inhibited in the presence of Fe^2+^ ions, while the activity of ALE-1 is even higher in the presence of these ions. Surprisingly, NaCl and KCl appeared to inhibit the activity of LytM at concentrations as low as 100 mM. The optimum pH values for enzyme activity are in the ranges of 6–9, 7–9, and 5–8 for lysostaphin, ALE-1, and LytM, respectively. The LytM exhibits unexpected thermostability, which is not observed in the case of the two other enzymes. The activity of LytM is stable for 15 min at 100 °C and is destroyed on heating for 30 min (Schindler and Schuhardt 1964; Sugai et al. 1997; Ramadurai et al. 1999). These details can be crucial due to its practical applications; for example, Sabala and coworkers using a newly developed model of chronic *S*. *aureus*-infected eczema observed evident lower activity of LytM in comparison to lysostaphin (Sabala et al. 2012).

The major autolysin of *S*. *aureus* is the AtlA protein (Oshida et al. 1995) that is quite similar in both sequence and domain organization to the AtlE, the predominant PH of *S*. *epidermidis* (Heilmann et al. 1997). Both of them are bifunctional, composed of an amidase domain and a glucosaminidase domain, which are separated by three repetitive sequences. The *atl* gene products undergo proteolytic processing, and as a result, two extracellular lytic enzymes can be found in the culture broth of both staphylococci. The amidase domain of AtlE was expressed in *E*. *coli* cells as a N-terminal His-tagged fusion protein and the product of expression exhibited staphylolytic activity. The analysis of the products of hydrolysis of *S*. *aureus* peptidoglycan revealed that the enzyme can be classified as a *N*-acetylmuramyl-l-alanine amidase (Biswas et al. 2006). These results clearly indicate that also the most important staphylococcal autolysins, derived from exogenous sources, could be used as staphylolytic agents. The same results can be expected in the case of other staphylococcal PHs.

### Bacteriophage lysins

The double-stranded DNA bacteriophages are released from bacterial cell host using a holins–endolysin system (Borysowski et al. 2006). Lysins are the enzymes targeting the integrity of the cell wall of the host and are designed to hydrolyze one of the four major bonds present in the peptidoglycan. Usually they do not have any signal sequences and are dependent on a second protein, called holins, to reach their substrate (Wang et al. 2000). The holins form pores in the inner membrane of the infected cells resulting in access of lysin to the peptidoglycan causing rapid cell lysis (Young and Blasi 1995). For phages, both the holins and the lysins are essential for host cell destruction. There are also some bacteripohages producing endolysins provided with a signal peptide. These enzymes are released from the cells of infected bacteria through the general secretion system (Park et al. 2007). However, when lysins of both groups are used as recombinant enzymes and applied exogenously, they rapidly lyse Gram-positive cells upon direct contact with peptidoglycan. The Gram-negative bacteria are more resistant to lysin activity because of the presence of an outer membrane (Loessner 2005; Fenton et al. 2010a). However, removing the outer membrane with specific compounds such as chloroform, EDTA, or detergents results in full sensitivity of these bacteria to the activity of lysins.

During the last decade, several interesting endolysins with bactericidal activity against staphylococci have been identified, isolated, and characterized. Some of them were tested as potential therapeutic agents or food preservatives. In general, *S*. *aureus* phage lysins display a multi-domain modular structure comprising of a C-terminal cell wall binding domain and two N-terminal catalytic domains. Examples include LysK, MV-L, phi11, and LysH5 (O’Flaherty et al. 2005; Donovan et al. 2006; Rashel et al. 2007; Obeso et al. 2008). One of the most promising seems to be the 495 aa endolysin LysK derived from staphylococcal phage K. O’Flaherty and coworkers cloned and expressed LysK in *Lactococcus lactis*. The obtained lactococcal lysates containing recombinant LysK were found to inhibit a range of different species of staphylococci isolated from bovine and human infection sources, including MRSA (O’Flaherty et al. 2005). The group of Becker et al. (2008) effectively expressed the C- and N-terminal His-tag version of the enzyme in *E*. *coli* cells. The authors found some problems with purification of N-His-LysK, which was co-purified with multiple smaller proteins, probably the products of degradation of the target protein, that were absent while full-length C-His-LysK was purified. The produced endolysin was found to exhibit highly bactericidal activity against *S*. *aureus* strain MRSA USA300. LysK construct with the C-His-LysK yielding a MIC of 32.85 μg/mL and N-His-LysK with a MIC of 80 μg/mL, in comparison with lysostaphin, inhibits the growth of the same strain at a concentration of 0.096 μg/mL. The enzyme maintained its activity at either 4 or −80 °C for up to 60 days. It was also revealed that lysostaphin and LysK (C-terminal His-tag version) act synergistically to kill MRSA. In the assay carried out by the group of Becker, two of the most effective conditions were at concentrations of 0.027 and 5.71 μg/mL and 0.018 and 11.43 μg/mL for lysostaphin and LysK, respectively, representing concentrations that correspond to 30 and 18 % and 16 and 33 % of each compound MIC, respectively (Becker et al. 2008). Detailed characteristic of the enzyme in terms of factors influencing its kinetic features has been presented by the group of Filatova et al. (2010). The authors also proposed the methods of stabilization of the enzyme. LysK incubated at 22 °C for 1 month loses over 90 % of its activity. The stability of the enzyme in the higher temperatures can be dramatically enhanced by the addition of low molecular weight polyoles (glycerol, sorbitol, sucrose, or trehalose) to the concentration over 50 % and Ca^2+^ ions to the concentration in the range of 1–5 mM (Filatova et al. 2010).

The LysK contains two enzymatic domains, one of which is an N-acetylmuramoyl-l-alanine amidase and the other a cysteine/histidine-dependent amidohydrolase/peptidase designated CHAP_k_. In 2009, two groups, Becker et al. (2009) and Horgan et al. (2009), published the results of their investigations aiming at the analysis of bactericidal activity of separated domains of LysK. The group of Becker revealed that the truncated version of LysK, containing just the CHAP domain, lyses *S*. *aureus* cells in zymogram analysis, plate lysis, and turbidity reduction assays but has no detectable activity in the MIC assay. In contrast, truncations harboring just the amidase lytic domain show faint activity in both the zymogram and turbidity reduction assays, but no detectable activity in either plate lysis or MIC assays. The most important observation of this investigation was that the fusion of the CHAP domain to the SH3b domain has near full-length LysK lytic activity, suggesting the need for a C-terminal binding domain. Both LysK and the CHAP-SH3b fusion were shown to lyse untreated *S*. *aureus* and the coagulase-negative strains (Becker et al. 2009). Interestingly, Horgan et al. (2009) revealed that separated CHAP domain (consisted of amino acids 1–156 of full-length LysK) was approximately 2-fold more active against live staphylococcal cells than the native enzyme. Additionally, the truncated enzyme was conveniently overexpressed in a highly soluble form (Horgan et al. 2009). Using the in vivo imaging system, it was also demonstrated that CHAP_k_ effectively eliminates *S*. *aureus* from the nares of artificially infected BALB/c mice (Fenton et al. 2010b). The lytic spectrum of the recombinant, effectively expressed in *E*. *coli* CHAP_k_ (10 mg from 1 L of bacterial culture), includes all staphylococcal species and also members of the genera *Micrococcus*, *Streptococcus*, *Nesterenkonia*, *Arthrobacter*, *Leuconostoc*, and *Carnobacterium*. The enzyme was active from pH 6 to 11, with an optimum activity at pH 9 and temperature varied from 5 to 40 °C, with an optimum activity at 15 °C, which can be a crucial in some applications (Fenton et al. 2011). The Hyglos GmbH (Regensburg, Germany) constructed a chimeric lysin containing an enzymatic active domain—cysteine and histidine-dependent aminopeptidase/hydrolase (CHAP) from LysK and cell wall binding domain from lysostaphin. The excellent activity of the chimeric enzyme, called PRF-119, was confirmed on large groups of MSSA (*n* = 398) and MRSA (*n* = 776) isolates. The MIC values were in the ranges 0.024–0.78 and 0.024–1.563 μg/mL, respectively (Idelevich et al. 2011).

Recently, the group of Jun et al. (2011) isolated endolysin SAL-1 the amino acid sequence of which differs from that of LysK at only three residues: isoleucine instead of valine at the 26th residue, glutamine instead of glutamic acid at the 114th residue, and histidine instead of glutamine at the 486th residue. In spite of the high degree of amino acid sequence similarity, the newly discovered phage endolysin SAL-1 has an approximately 2-fold lower MIC against several *S*. *aureus* strains and higher bacterial cell wall-hydrolyzing activity than LysK. The authors constructed several mutants of SAL-1 and revealed that the amino acid residue change contributing the most to this enhanced enzymatic activity is a change from glutamic acid to glutamine at the 114th residue. It would be interesting to check the activity of truncated enzyme containing only the CHAP_k_ domain with modified amino acid residue at position 114. The enzyme has been effectively expressed in *E*. *coli* cells as C-terminal His-tagged protein and should be also considered as a potential antistaphylococcal agent.

Another interesting antistaphylococcal lysine assigned MV-L has been isolated from the phage øMR11. The enzyme consists of 481 amino acid residues and, similar to other lysins, is composed of three functional domains: exhibiting endopeptidase activity (amino acid residues 15 to 120), amidase (amino acid residues 199 to 322), and cell wall recognition domain (amino acid residues 323 to 481). The lysin rapidly and completely lysed cells of a number of *S*. *aureus* strains tested, including methicillin-resistant *S*. *aureus* (MRSA) and vancomycin-resistant *S*. *aureus* and a subset of vancomycin-intermediate *S*. *aureus* (VISA) in growing conditions. MV-l-mediated killing is specific to *S*. *aureus* and not to other species, except for *S*. *simulans*. MV-L exerted its staphylocidal effect synergistically with glycopeptide antibiotics against VISA. The lysin efficiently eliminated MRSA that had been artificially inoculated into the nares of mice. The intraperitoneal administration of MV-L also protected mice against MRSA septic death, without any harmful effects (Rashel et al. 2007).

The in vivo antistaphylococcal activity has been also confirmed in the case of lysin ClyS constructed by Daniel et al. (2010). The chimeric lysin was obtained by fusing the N-terminal endopeptidase domain of the *S*. *aureus* Twort phage lysin with the C-terminal cell wall-targeting domain from another *S*. *aureus* phage lysin (phiNM3), which displayed *Staphylococcus*-specific binding. The non-His-tagged ClyS obtained in the expression host, *E*. *coli*, lysed VISA, MSSA as well as MRSA strains of *S*. *aureus* in vitro. ClyS was also active against other staphylococci: *S*. *epidermidis*, including the biofilm-forming strain RP62A, *S*. *simulans*, and *S*. *sciuri* subsp. *sciuri*, did not exhibit activity against other Gram-positive and Gram-negative bacteria, including representatives of group A, B, C, and E streptococci, the oral streptococcal species *S*. *gordonii* and *S*. *salivarius* as well as *Streptococcus uberis*, *Bacillus cereus*, *P. aeruginosa*, and *E*. *coli*. But the most interesting and promising results were obtained in mouse nasal decolonization model. The 2-log reduction in the viability of MRSA cells was seen in 1 h following a single treatment with ClyS. Single intraperitoneal dose of ClyS also protected against death caused by MRSA in a mouse septicemia model. ClyS showed a typical pattern of synergistic interactions with both vancomycin and oxacillin in vitro. More importantly, ClyS and oxacillin at doses that were not protective individually, in combination, protected synergistically against MRSA septic death in a mouse model (Daniel et al. 2010). In a mouse model of skin colonization/infection with *S*. *aureus*, the ClyS was shown to be more effective than mupirocin. The lysin eradicated a significantly greater number of methicillin-susceptible *S*. *aureus* and methicillin-resistant *S*. *aureus* bacteria; the 3- and 2-log reductions were observed for ClyS and mupirocin, respectively, and no influence of produced antibodies against the lysine activity was observed (Pastagia et al. 2011). In vitro and in vivo activity was also confirmed for staphylococcal peptidoglycan hydrolase LysGH15, which was first isolated from bacteriophage GH15. In animal experiments, a single intraperitoneal injection of LysGH15 (50 μg) administered 1 h after MRSA injections at double the minimum lethal dose was sufficient to protect mice (*P* < 0.01). Bacteremia in unprotected mice reached colony counts of about 10^7^ CFU/mL within 3.5 h after challenge, whereas the mean colony count in lysin-protected mice was less than 10^4^ CFU/mL or even became undetectable (Gu et al. 2011a). It was also found that the enzyme alleviates inflammation induced by infection with lethal MRSA (Gu et al. 2011b).

High lytic potential towards *S*. *aureus* was also noticed in the case of bacteriophage P-27/HP from sewage water. The isolated endolysin produced by this phage assigned with the same numerical code exhibited maximum in vitro lytic activity at temperatures between 35 and 40 °C and pH 7.0. In vivo experiments revealed considerable (99.9 %) elimination of *S*. *aureus* 27/HP from spleens of endolysin-treated mice and had saved them from death due to infection (Gupta and Prasad 2011a, b).

In the case of many interesting lysins, such as protein 17 of the bacteriophage P68, a bactericidal activity towards *S*. *aureus* has been confirmed only using the in vitro tests. The enzyme was tested on 35 clinical isolates of *S*. *aureus*, 50 % of which were oxacillin-resistant. The purified lysin showed a broader antimicrobial spectrum, compared to the host range of phage P68. The phage lysed only 11 of all clinical strains, whereas protein 17 displayed activity against 26 strains. This suggested that the bacterial receptor required for phage attachment differs from the ligand required for protein 17 binding (Takac and Blasi 2005). High thermostability was noticed in the case of HydH5 lysin, produced by *S*. *aureus* bacteriophage vB_SauS-phiIPLA88 (phiIPLA88). The enzyme retained 72 % of its activity after 5 min at 100 °C (Rodríguez et al. 2011).

Several investigations confirmed potential usefulness of some bacteriophage lysins as food preservatives. The purified, recombinant LysH5 lysin, from bacteriophage øH5 was able to kill rapidly *S*. *aureus* growing in pasteurized milk and the pathogen was not detected after 4 h of incubation at 37 °C (Obeso et al. 2008). Additionally, the results of the investigation carried out by García et al. (2009) suggest that activity of lysin produced by øH5 can be enhanced by its combination with other peptidoglycan hydrolases. A mixture of phages øH5 and øA72 was more effective than each single phage. The mixture of phages effectively inhibited *S*. *aureus* in UHT and pasteurized whole fat milk. However, the phages producing lysins were less active in semi-skimmed raw milk, and little inhibition was achieved in whole, raw milk. Killing of *S*. *aureus* was observed at room temperature and at 37 °C, but not at refrigeration temperature (García et al. 2009). The same authors tested LysH5, the endolysin encoded by the staphylococcal bacteriophage phi-SauS-IPLA88. When LysH5 was combined with nisin, a strong synergistic effect was observed. The MIC of nisin and LysH5 were reduced 64- and 16-fold, respectively, as determined in checkerboard microtiter tests. In addition, nisin enhanced 8-fold the lytic activity of LysH5 on cell suspensions. Importantly, the synergy observed in vitro was confirmed in challenge assays in pasteurized milk contaminated with *S*. *aureus* Sa9. Clearance of the pathogen was achieved only by the combined activity of both antimicrobials (García et al. 2010). Recently, Rodriguez-Rubio and coworkers constructed a strain of *L. lactis* producing recombinant, biologically active LysH5. Despite the fact that almost 80 % of protein is retained in the cell and only about 20 % is secreted, the strain seems to be a promising microorganism inhibiting the growth of *S*. *aureus* in fermented food products (Rodriguez-Rubio et al. 2012). Donovan et al. (2006) demonstrated that the endolysin ø11 exhibits bactericidal activity at the physiological pH and calcium concentrations found in milk towards not only *S*. *aureus*, but also several species of mastitis-causing CoNS, which makes it not only a potential milk food product preservative, but also a promising anti-mastitis agent. Additionally, Sass and Bierbaum (2007) revealed that ø11 endolysin is able to hydrolyze staphylococcal biofilm. It is important to remember that nature is still a reservoir of probably a great number of staphylococcal bacteriophages which are not yet recognized. Recently, Kwiatek et al. (2012) isolated and characterized of a novel virulent bacteriophage (MSA6) from a cow with mastitis. Isolated phage was capable of infecting a wide spectrum of staphylococcal strains of both human and bovine origin; however, the sequence of the lysin is still not known.

The number of publications concerning isolation, characterization, and possibilities of the application of bacteriophage lysins as antistaphylococcal agents rapidly increased during the last 5 years. The presented results are very promising. Much effort is required in elaboration of big scale technologies of their production and testing of their toxicity, allergenicity, and eventually influence on the components of natural flora of the human and animal body. However, it can be assumed with high probability that in the near future, this group of proteins will be used as food preservatives and in the therapy of infections caused by staphylococci.

## Conclusions

*S*. *aureus* is a major human and animal pathogen responsible for a number of serious and sometimes fatal infections. The emergence of antibiotic-resistant *S*. *aureus* strains has resulted in significant treatment difficulties. Therefore, there is an urgent need to develop novel antibacterial agents to control this pathogen. One of the most promising solutions of the problem could be the application of enzymes hydrolyzing cell wall peptidoglycan of *S*. *aureus*. The most important advantages of these enzymes are (1) high specificity; in fact, most of the PHs described above are completely inactive toward other than *S*. *aureus* groups of bacteria. As a result, they are considered as safe for natural microbiota of the infected organism; moreover, they can be applied for the protection of fermented food products. On the other hand, some of the PHs described above are characterized by broader spectrum including coagulase-negative staphylococci—pathogens of growing importance and even bacteria of other origin. As a consequence, it is possible to select the most appropriate enzyme of particular therapeutic or technological problem which has to be solved. (2) Most investigations, carried out up to date, classified these substances as safe, with low toxicity and immunogenicity; (3) most of them have been successfully produced using heterologous *E*. *coli* expression systems characterized with high efficiency; (4) some of them act synergistically with other antimicrobial agents including antibiotics; (5) some of the PHs described, especially of bacteriophage origin, are composed of two catalytic domains, which recognize and hydrolyze two different bonds within the peptidoglycan. It evidently reduces the possibility of the creation of resistant strains; (6) they are active even against multidrug-resistant strains and can be used for eradication of bacteria growing in biofilm. The summary characteristic of most promising antistaphylococcal PHs is presented in Table 1.

**Table 1:** Summary of the best characterized antistaphylococcal peptidoglycan hydrolases

Enzyme	Source organism	Enzyme activity	Spectrum	Host for heterologous expression	Synergistic interactions
Lysostaphin	*S*. *simulans* biovar *staphylolyticus*	Interpeptide bridge endopeptidases	*S*. *aureus*	*E*. *coli*, *L*. *casei*, *L*. *curvatus*, *L*. *sake*, *L*. *lactis*	Antibiotics, antimicrobial peptides, other peptidoglycan hydrolases, tea tree oil
LasA	*P*. *aeruginosa*	Interpeptide bridge endopeptidases	*S*. *aureus* and other staphylococci	–	ND
ALE-1	*S*. *capitis* EPK1	Interpeptide bridge endopeptidases	*S*. *aureus*	*E*. *coli*	ND
LytM	*S*. *aureus*	Interpeptide bridge endopeptidases	*S*. *aureus*	*E*. *coli*	ND
AtlA	*S*. *aureus*	Amidase and glucosaminidase	*S*. *aureus*		ND
AtlE	*S*. *epidermidis*	Amidase and glucosaminidase	*S*. *epidermidis*	*E*. *coli*	ND
LysK	Phage K	N-acetylmuramoyl-l-alanine amidase	*S*. *aureus*, other staphylococci, *Micrococcus*, *Streptococcus*, *Nesterenkonia*, *Arthrobacter*, *Leuconostoc*, and *Carnobacterium*	*E*. *coli*, *L*. *lactis*	Lysostaphin
		Cysteine/histidine-dependent amidohydrolase/peptidase			
SAL-1	Bacteriophage SAP-1	N-acetylmuramoyl-l-alanine amidase	*S*. *aureus*, *S*. *saprophyticus*, *S*. *epidermidis*	*E*. *coli*	ND
		Cysteine/histidine-dependent amidohydrolase/peptidase			
MV-L	Bacteriophage phi MR11	Endopeptidase and amidase	*S*. *aureus*, *S*. *simulans*	*E*. *coli*	Glycopeptides antibiotics
ClyS	Constructed^a^	Endopeptidase	*S*. *aureus* and other staphylococci	*E*. *coli*	Vancomycin and oxacillin
LysH5	Bacteriophage phi-SauIPLA88	ND	*S*. *aureus*, lack information about other bacteria	*L*. *lactis*	Nisin

*ND* not determined

^a^Details in the text

Obviously, it is necessary to remember about several limitations which have to be solved before using PHs as therapeutic agents or food preservatives. The most important issues, which at the current state of knowledge preclude clinical or industrial application of these enzymes, have been discussed below. It should be noted that in this respect only lysostaphin has been tested at a sufficient level; however, it can be expected that trials of practical application of other PHs will result in the same problems and limitations which have been observed for lysostaphin. We have not only presented the most important limitations but also discussed the methods of overcoming them.

1. However, at the current state of knowledge, the PHs, including lysostaphin, are generally recognized as safe, and it is necessary to intensify the studies on toxicity and immunogenicity coming from using these enzymes. The results of few confirmed production of anti-lysostaphin antibodies occur in many different animal models; however, their production did not significantly affect the activity of the enzyme. Using a rabbit model of ocular MRSA infection, Dajcs et al. (2002) achieved very good effects of eradication of bacteria from both immunized and non-immune rabbits. Additionally, no adverse reactions were observed due to administration of lysostaphin to immunized rabbits. No allergic reactions nor other deleterious symptoms in the host animal were obtained in the effect of intramammary infusion of 2–3 g (18–21 infusions) of recombinant lysostaphin used as a *S*. *aureus* bovine mastitis agent (Daley and Oldham 1992). However, the results of preliminary research are promising, and there is no doubt that additional analyses according to regulatory requirements are necessary.
2. All of these enzymes except for lysostaphin are not available commercially. The possibilities of production of most PHs characterized above using efficient heterologous *E*. *coli* expression systems have been described. However, they are produced only in a small scale for research needs. To date, only the industrial scale production technology of lysostaphin has been developed. Using a *L. lactis* nisin-controlled system, Mierau et al. (2005a) optimized conditions of enzyme production at the 3,000-L scale and obtained a final yield of lysostaphin after purification about 120 g per batch. The same authors (Mierau et al. 2005b) revealed also that in the small scale (1 L fermentor), the efficiency of the expression system can be increased to the level of 300 mg/L. It is necessary to develop large-scale production technologies of other promising PHs, which require much effort in each case. First of all, it would be necessary to use other than *E*. *coli* expression systems for their production. The most popular, commercially available *E*. *coli* systems of recombinant protein production such as pET or pBAD are usually useless for industrial scale protein production. Low level of expression, instability of strains producing proteins, incorrect folding of proteins (e.g., creation of incorrect disulfide bonds and production of the protein in the form of inclusion bodies), strong dependence of level of production, and conditions of fermentation are the most commonly observed limitations. Large-scale production technologies of many proteins have been developed using yeast expression systems, especially *Picha pastoris* and *Sachcaromyces cerevisiae*. Surprisingly to date, there are no information about trials of heterologous production of any PHs in yeast cells. There is no doubt that intense research on this subject should be done as there is a high possibility of selection expression host for PH production better than *E*. *coli*. Enhanced availability of the proteins will reduce their prices, which can be crucial not only for its application in medicine, veterinary, and food technology, but also for further tests of their properties during preclinical and clinical research. At the moment, the price of 1 and 15 mg of lysostaphin produced by a leading supplier is 100 and 800 €, respectively, which can be a problem even for its application as a therapeutic agent and is completely unacceptable for its use as a food preservative.
3. Another challenge is to develop the technologies of formulation which could guarantee a long-term stability of the enzymes without loss of their activity.
4. The effectiveness of eradication of the bacteria from the infected tissue will also depend on the method of protein administration. The importance of both issues—formulation and administration—has been partially investigated by the group of Walsh et al. (2003). The authors improved the pharmacokinetics of lysostaphin by its PEGylation with branched 40 kDa PEG. According to in vitro tests, the PEGylated enzyme is characterized by lower activity in comparison to the unconjugated enzyme. The compensate of the partial decrease of activity of PEGylated enzyme was an evident increase of its half-life in mice blood serum. PEGylated lysostaphin injected intravenously had a drug half-life in serum of up to 24 h and resulted in much higher plasma drug concentrations than an equal dose of unconjugated lysostaphin, which had a half-life of less than 1 h. Additionally, the PEGylation resulted in a greater than 10-fold reduction in antibody affinity in comparison to unconjugated protein. These results significantly enhance the therapeutic value of lysostaphin and other PHs as intravenous antistaphylococcal agent.
5. The natural consequence of treatment of bacteria with bactericidal or bacteriostatic agent is selection of mutants resistant to their activity. The problem is especially common when the therapy is carried out for a long period of time and only with using one agent. Selection of *S*. *aureus* mutants with reduced susceptibility to lysostaphin has been observed by several authors. The selected mutants are characterized with mutations within *fem* or *lyrA* operon which resulted in modification of amino acid composition of interpeptide bridges-the molecular target of lysostaphin and also several other PHs described in this review (de Jonge et al. 1993; Boyle-Vavra et al. 2001; Grundling et al. 2006). In fact, this limitation can be surmounted by using therapies that contain multiple antimicrobial agents. To date, several successful trials of lysostaphin containing combination treatments have been described. The enzyme shown to be synergistically bactericidal in combination with various agents, including antibiotics (Polak et al. 1993; Climo et al. 1998; Kiri et al. 2002; Walencka et al. 2006), antimicrobial peptides (Desbois and Coote 2011), and even some natural products, e.g., tea tree oil (LaPlante 2007). The similar effects have been observed in the case of several other PHs, e.g., ClyS, which showed synergistic interactions with vancomycin and oxacillin. It may be assumed with a high probability that future research will lead to the discovery of promising combinations of PHs with other antimicrobial compounds. The benefit of the combinations of various antimicrobial agents is not only limited to the development of resistance phenomenon but also usage of lower doses of agents essential for successful therapy.

PHs seem to be promising antistaphylococcal agents, which is confirmed by a number of investigations whose results we have presented in this review. The discussions presented above clearly indicate that it is possible to solve most of the problems which currently preclude application of these enzymes as therapeutic agents in human and animal infections or as a food preservative. However, still much effort on this subject is required. The main goals of the authors of this review were to highlight the therapeutic and prophylactic potential of antistaphylococcal PHs and indicate the most crucial limitations of their practical application at the current state of knowledge as well as encourage readers to take any form of effort which could result in practical usage of these enzymes in medicine, veterinary, and the food industry in the nearest future.
